# A mixed methods systematic review of mental health self-care strategies for Arabic-speaking refugees and migrants

**DOI:** 10.1186/s12889-023-17395-9

**Published:** 2023-12-20

**Authors:** Deena Mehjabeen, Ilse Blignault, Perjan Hashim Taha, Nicola Reavley, Shameran Slewa-Younan

**Affiliations:** 1 Translational Health Research Institute, Western Sydney University, Campbelltown, NSW Australia; 2 College of Medicine, University of Duhok, Duhok, Iraq; 3 School of Medicine, Western Sydney University, Campbelltown, NSW Australia; 4 Centre for Mental Health, Melbourne School of Population and Global Health, University of Melbourne, Melbourne, VIC Australia

**Keywords:** Mental health, Self-care, Coping, Arabic-speaking, Refugee, Migrant, Global health, Systematic review

## Abstract

**Background:**

Self-care strategies can improve mental health and wellbeing, however, the evidence on preferred strategies among Arabic-speaking refugees and migrants is unclear. This mixed methods systematic review aimed to identify and synthesise the global research on mental health self-care strategies used by these populations.

**Methods:**

English and Arabic language studies reporting on positive mental health self-care strategies to address symptoms of posttraumatic stress disorder, generalised anxiety and depression in the target populations were identified by systematically searching eight electronic databases and grey literature. Studies were deemed eligible if they were published from 2000 onwards and included Arabic-speaking migrants, refugees or asylum seekers aged 12 years and above. A narrative synthesis of study characteristics and relevant key findings was undertaken. The review protocol was registered on PROSPERO (registration number CRD42021265456).

**Results:**

Fifty-nine records reporting 57 studies were identified, the majority appearing after 2019. There were 37 intervention studies that incorporated a self-care component and 20 observational studies that reported on self-generated self-care practices. Across both study types, four broad groups of mental health self-care were identified—social, psychological, religious/spiritual, and other (e.g., expressive arts and exercise). Psychological strategies were the most reported self-care practice overall and featured in all intervention studies. Religious/spiritual and social strategies were more common in the observational studies. Intervention studies in diverse settings reported statistical improvements on a range of outcome measures. Observational studies reported a range of individual and community benefits. Linguistic, cultural and religious considerations, inherent in the observational studies, were variably addressed in the individual and group interventions.

**Conclusion:**

Overall, study participants experienced self-care as helpful although some encountered challenges in practicing their preferred strategies. Further research on mental health self-care strategies among Arabic-speaking refugees and migrants is needed in Western resettlement countries to guide mental health service delivery and primary healthcare initiatives for new arrivals and in transit countries.

**Supplementary Information:**

The online version contains supplementary material available at 10.1186/s12889-023-17395-9.

## Introduction

The world is witnessing an unprecedented rise in the number of international migrants, refugees, and asylum seekers [1]. Forced migration is a growing concern and has been recognised as a major global health issue [2, 3]. By mid-2023, approximately 110 million individuals were forcibly displaced from their homes due to war, conflict or persecution, with 36.4 million recognised as refugees [4]. Many originate from countries where Arabic is widely spoken, such as Syria, accounting for the highest worldwide refugee population at 6.5 million [4].

Refugees and asylum seekers arrive in host countries carrying the psychological burden of their journeys [5]. Systematic reviews and meta-analyses indicate higher prevalence of mental disorders among refugees and asylum seekers than in the general population [6–9]. For example, a recent systematic review and meta-analysis of global refugee populations reported prevalence rates of 31.5% and 31% for depression and posttraumatic stress disorder (PTSD) respectively, compared with 12% and 3.9% for the general population [10]. Among adult Syrian refugees resettled in 10 countries, 43% had PTSD symptoms, 40% had depression and 26% had generalised anxiety disorder [11]. Overall, they were over 10 times more likely to develop PTSD and other mental disorders than the general population [11]. Adolescent refugees also experience challenges which can lead to increased prevalence of PTSD, depression and anxiety [12–14].

Psychological distress often exacerbates upon arrival in the host country due to legal and economic barriers, language barriers, discrimination and acculturation stress [15–17]. These stressors contribute to elevated risk of PTSD and depression among refugees and asylum seekers [18–20]. Migrants who move to another country for better economic, social or educational prospects face similar resettlement stressors, increasing their risk of mental disorders [21, 22]. Many experience discrimination and social exclusion, difficulties in finding work according to their education level and acculturation and adaptation stressors [23–25].

Given such findings, it is of concern that resettled refugees and migrants are significantly less likely to seek professional help or utilise mental health services than the general population [23, 26]. Within Western countries, commonly reported barriers to appropriate and timely treatment-seeking include language limitations, low mental health literacy, healthcare costs, uncertain migration status, unstable housing, limited transportation, poor understanding of healthcare services, discrimination from healthcare professionals and distrust of authority [27–34].

With limited access to culturally appropriate professional mental health services, those with, or at risk of, mental disorders may rely on self-care, self-help or self-management strategies as coping mechanisms. Such strategies can be practiced by individuals to manage or overcome their psychosocial distress with or without professional input [35–37]. ‘Self-care’ has become the preferred term in the global health literature. It is defined by the World Health Organization (WHO) as ‘the ability of individuals, families and communities to promote health, prevent disease, maintain health and cope with illness and disability with or without the support of a health worker’ [38, p.2]. At the base of the WHO service pyramid model, self-care is the foundation for all other forms of care and is critical to achieving optimal mental health [39]. The WHO model highlights the active role individuals with mental disorders can play in caring for themselves with the support of family and community [40]. Studies show that people with mild depression symptoms find informal self-care (without professional guidance) helpful and that such approaches can be cost-effective in reducing symptoms of depression and other mental disorders [41–43].

Currently, a comprehensive understanding of preferred mental health self-care strategies among Arabic-speaking refugees and migrants is lacking. This systematic review, which encompasses self-care strategies used both within people’s everyday lives and as part of therapeutic interventions, will have implications for mental health programs and service delivery, and for future research. At the individual level, self-care strategies can empower people to look after their mental health. At the service level, self-care interventions (therapeutic tools that support self-care practices) can offer cost-effective mental health and wellbeing outcomes, optimising healthcare services and resources [38]. This systematic review aimed to identify and examine the evidence for self-care strategies in these globally significant populations. The primary question was ‘What are the self-care strategies used to mitigate mental health issues within Arabic-speaking refugee and migrant populations globally?’ Related sub-questions were (i) What is the evidence for the effectiveness (presented as measures of outcome) of the identified strategies? (ii) What is the evidence for the cultural appropriateness of the identified strategies? and (iii) What positive and negative experiences are associated with the identified strategies?

## Materials and methods

The Preferred Reporting Items for Systematic Reviews and Meta-Analyses (PRISMA) guidance was followed throughout this review [44]. The review protocol was registered on PROSPERO (registration number CRD42021265456) [45]. It is important to note that, although the term ‘self-help’ was used in the protocol title, the term ‘self-care’ as defined by the WHO is used here [38, 45].

### Search strategy and selection criteria

Searches for peer-reviewed studies published from January 2000 to April 2023 were undertaken in eight digital databases: Ovid MEDLINE, Scopus, EMBASE, CINAHL, Web of Science, PTSDpubs (formerly known as PILOTS), PsycINFO, and Dar Al Mandumah (a set of Arabic databases). Non-indexed journals were searched using the Ulrichsweb database. Search terms were searched in titles, abstracts, and keywords. Supplementary searches included registered clinical trials, reference lists of included full-text articles and Google Scholar. We searched the web for non-government organisational reports and unpublished theses using Trove (Australian thesis database) and EThOS (British Library thesis database). The search queries were formulated to achieve all relevant results on self-care strategies and mental disorders (including all their term variants and most common synonyms) among Arabic-speaking refugees and migrants in both transit and resettlement countries. Specific search strategies are detailed in the protocol [45].

### Inclusion criteria

Studies (quantitative, qualitative and mixed methods) were included if published in indexed or non-indexed journals in English or Arabic and focused on Arabic-speaking refugees, asylum seekers or migrants aged 12 years and older in any country, including countries of asylum/transit and resettlement. Studies were required to report mental health self-care strategies that address PTSD, depression, generalised anxiety disorder and panic disorder (common disorders in the target population). Psychotherapeutic interventions delivered by health professionals but having self-care components that could be applied by an individual independently (without professional involvement) were also included. We considered registered clinical trials and unpublished (grey) literature such as organisational and technical reports and dissertations.

### Exclusion criteria

Reviews, editorials, commentaries, letters to the editor, newsletters, newspapers, magazine articles, and books or book chapters were excluded. Studies on Arabic-speaking refugees, asylum seekers and migrants aged below 12 years and non-Arabic speakers were excluded. Studies that exclusively reported the perspectives of those providing formal support (e.g., mental health professionals, migrant support workers and refugee resettlement workers), prevalence studies, and research focusing on interventions for physical health conditions or social issues were excluded.

### Study selection

Systematic search results were first exported into Endnote to remove duplicates and then into Covidence (https://www.covidence.org/), an online screening and data extraction tool for systematic reviews. For English language articles, the first author and primary reviewer (DM) screened all titles and abstracts for eligibility. Full-text articles were double screened by two authors (DM and SSY) and discrepancies resolved by a third (IB). DM, SSY and IB double screened the grey literature titles and abstracts, and full texts. For Arabic language articles and dissertations, PHT performed the title-abstract and full-text screening. PHT also made English translations of the titles and abstracts, which were double screened by DM, SSY and IB.

### Quality assessment

Quality assessment was undertaken using the Joanna Briggs Institute (JBI) Critical Appraisal Tools (https://jbi.global/critical-appraisal-tools) relevant to the study design. The JBI tools are study-specific for quantitative (e.g., randomised controlled trials or RCTs, quasi-experimental studies and cross-sectional studies) and qualitative research [46]. For English language articles, two authors (DM and SSY) independently conducted the quality appraisal of the studies with a third (IB) involved in resolving disagreements. For articles in Arabic, PHT performed the quality appraisal. Grey literature was appraised using the JBI tools (independently undertaken by DM, SSY and IB for English language studies and PHT for Arabic language studies). For the RCT tool with a possible total score of thirteen, scores of 7 or below were deemed low quality, 8–10 as medium, and 11 and above as high. For the cross-sectional tool, with a possible total of 8, scores of four or below were deemed low quality, 5–6 as medium, and 7 and above as high. For the quasi-experimental (possible total 9) and qualitative (possible total 10) tools, scores of five or below were deemed low quality, 6–7 as medium, 8 and above were deemed as high. No studies were excluded due to their quality.

### Data extraction and synthesis

Relevant data were extracted by DM (English records) or PHT (Arabic). They included study characteristics, nature of self-care strategies, duration and mode of delivery, outcome measures, cultural appropriateness, and participant experience where reported. Our systematic review was designed to answer several interrelated questions. A narrative synthesis was undertaken due to the heterogeneity of study designs, which precluded meta-analysis. Narrative synthesis refers to ‘an approach to the systematic review and synthesis of findings from multiple studies that relies primarily on the use of words and text to summarise and explain the findings of the synthesis’ [47, p.5]. We focused on positive self-care strategies expected to have a beneficial effect on mental health and wellbeing, while excluding negative activities such as the consumption of tobacco, alcohol or drugs [48–55].

## Results

### Study characteristics

**Fig. 1:**
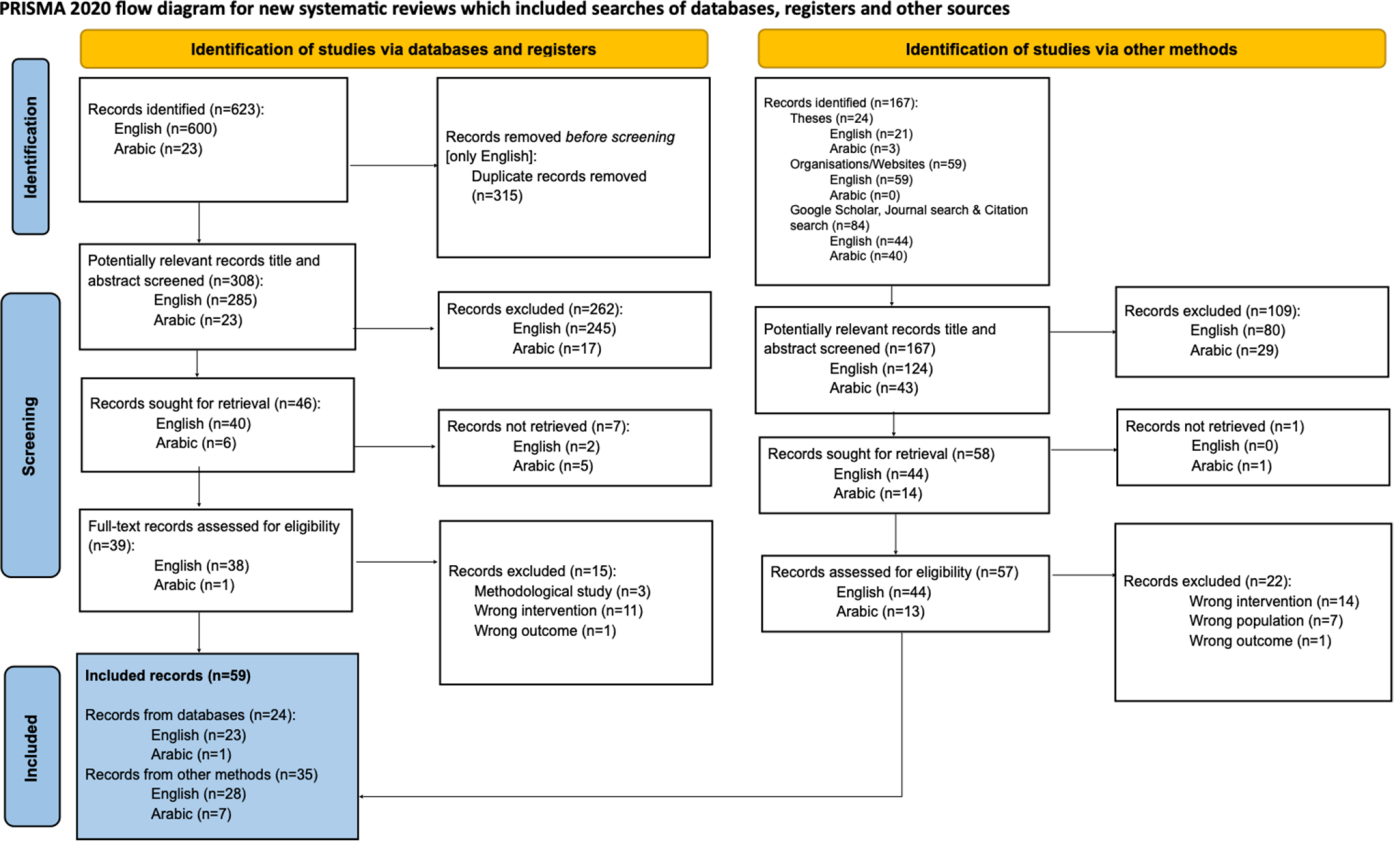
PRISMA flow diagram of the number of records identified, screened, assessed for eligibility and included in this review

**Fig. 2:**
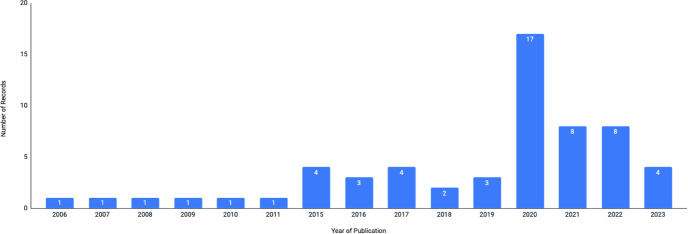
Number of included records by year of publication

The review flowchart according to PRISMA [56] is presented in Fig. 1. Database searches yielded 623 records, with an additional 167 records identified through organisational reports, theses and manual citation searches. After removing duplicates and screening records for eligibility, we identified 96 records for full text screening. Fifty-nine records were eligible for inclusion. As Fig. 2 shows, most appeared after 2019 (n = 37). Five English language articles were authored by members of the research team (SSY, IB). One journal article and a dissertation discussed the same study [48, 49] and two articles [57, 58] reporting an intervention and the follow-up were considered as one study. The 57 discrete studies were categorised as observational (n = 20) or intervention (n = 37). Of the 20 observational studies, 14 used qualitative methods (semi-structured interviews, focus group discussions and ethnography), five used mixed methods and one was quantitative. Of the 37 intervention studies, 25 were quantitative (randomised controlled trials and quasi-experimental studies), eight were mixed methods and four were qualitative.

Table 1 summarises the characteristics of the included studies. A detailed description of each study is provided in S1 File. Studies were classified as high, middle-or low-income countries using the World Bank classification [59]. Fourteen observational studies were conducted in high-income countries (HICs) including Germany (n = 5) and United States (n = 4) [48, 49, 54, 55, 60–70]. Eighteen intervention studies were conducted in upper middle-income countries (UMICs) [57, 58, 71–87], majority in Jordan (n = 14). Sudan was the only low-income country (LIC). For this review, we considered HICs as resettlement countries. As shown in Table 1, all but one of the observational studies were deemed high quality. Of the 37 intervention studies, 25 were deemed as high quality and 12 as medium quality.

**Table 1:** Characteristics of included studies by study type

Author (Year)	Study Location	Study Type & Methods	Target Population	Sample Characteristics	JBI Quality Appraisal
**Observational Studies (n = 20)**					
Al-Ajarma (2010)	United States (HIC)	Qualitative: Narrative inquiry and semi-structured interviews	Palestinian graduate students	**n** = 7 **Age**: 28-44 years **Gender**: 43% females **Religion**: not specified **Education**: 57% university **Country of origin**: 100% Palestine	Qual = 9/10 High
Alhaddad et al. (2021)	Germany (HIC)	Qualitative: Semi-structured interviews	Newly arrived Syrian and Iraqi refugee youths	**n** = 20 **Age**: 14–18 years (*M* = 16, *SD* = 1.7) **Gender**: 35% females **Religion**: 75% Islam, 10% Izidi, 15% none **Education**: 65% regular classes, 35% welcome classes **Country of origin**: 70% Syria, 30% Iraq	Qual = 9/10 High
Boswall & Akash (2015)	Jordan (UMIC)	Qualitative: In-depth ethnography	Syrian women and adolescent girls	**n** = 50 **Age**: 14–66 years **Gender**: 100% females **Religion**: 100% religious Muslims **Education**: incomplete education levels **Country of origin**: 100% Syria	Qual = 9/10 High
Chaaya et al. (2007)	Lebanon (UMIC)	Mixed Methods: questionnaire and interviews	Older adults in three poor suburbs of Beirut, one of which was a Palestinian refugee camp	**n** = 246 (Palestinian refugees) **Age**: ≥60 years **Gender**: 54% females **Religion**: 100% Muslims **Education**: 40% literate **Country of origin**: 95% Palestine, 5% Lebanon	Cross-sectional = 8/8 High Qual = 9/10 High
Darychuk & Jackson (2015)	West Bank/Palestine (LMIC)	Qualitative: Semi-structured interviews	Palestinian women in West Bank camps	**n** = 31 **Age**: 22–48 years (*M = 35*) **Gender**: 100% females **Religion**: not specified **Education**: not specified **Country of origin**: 100% Palestine	Qual = 10/10 High
Ferguson (2015)	United States (HIC)	Qualitative: Semi-structured interviews	Iraqi refugee men	**n** = 10 **Age**: 18–60 years **Gender**: 0% females **Religion**: 50% Chaldeans, 50% Muslims **Education**: 10% primary, 90% post primary **Country of origin**: 100% Iraq	Qual = 10/10 High
International Medical Corps (2017)	Jordan (UMIC)	Mixed Methods: Questionnaire, key informant interviews (KII) and focus group discussions (FGDs)	Syrian refugees in 11 cities and 2 camps and Jordanian nationals in same cities	**n** = 6,375 **Age**: ≥18 years (*M = 34, SD = 11*) **Gender**: 59% females (Urban); 39% females (Camp) **Religion**: not specified **Education**: 90% formal (Urban); 79% formal education (Camp) **Country of origin**: Syria	Cross-sectional = 8/8 High Qual = 9/10 High
International Medical Corps Lebanon (2011)	Lebanon (UMIC)	Qualitative: Semi-structured interviews	Forcibly displaced Syrians at Lebanon-Syria border	**n** = 100 **Age**: 32 years (mean age only) **Gender**: 64% females **Religion**: 100% Sunni Muslims **Education**: 18% illiterate, 79% elementary & high school **Country of origin**: 100% Syria	Qual = 7/10 Medium
Irfaeya (2006) Irfaeya et al. (2008)	Germany (HIC)	Mixed Methods: Survey, KII and FGD	Arab migrant women in Cologne	**n** = 116 **Age**: ≥18 years *(M = 32*) **Gender**: 100% females **Religion**: 100% Muslims **Education**: 8% illiterate, 68% school, 54% post school **Country of origin**: 31% Morocco, 23% other, 12% Lebanon, 9% Germany, 9% Tunisia, 9% Iraq, 8% Syria	Cross-sectional = 6/8 Medium Qual = 9/10 High
Jamil (2020)	Canada (HIC)	Mixed Methods: Questionnaire and semi-structured interviews	First or 1.5 generation adult Arab immigrants	***Quantitative***: **n** = 172 **Age**: ≥18 years (*M = 30, SD = 13, range = 18–65*) **Gender**: 58% females **Religion**: 67% Muslims, 33% Christians **Education**: 21% high school, 16% college, 49% university, 15% other **Country of origin**: 92% Middle Eastern, 9% North and East African ***Qualitative***: **n** = 11 **Age**: ≥18 years (*M = 29, SD = 10, range = 19–57*) **Gender**: 55% females **Religion**: 82% Muslims, 9% Christians **Education**: 9% high school, 18% college, 18% university, 18% other **Country of origin**: 99% Middle Eastern	Cross-sectional = 8/8 High Qual = 10/10 High
Kadri (2009)	New Zealand (HIC)	Qualitative: Semi-structured interviews	Arabic-speaking Muslim refugees from Arab/Muslim countries	**n** = 31 **Age**: 16–52 years **Gender**: 52% females **Religion**: 100% religious Muslims **Education**: 3% no school, 71% school, 26% university **Country of origin**: 74.1% Iraq, 9.7% Kuwait, 6.5% Sudan, 6.5% Somalia, 3.2% Tunisia	Qual = 10/10 High
Keshavarzi (2018)	Canada (HIC)	Qualitative: Semi-structured individual interviews	Recently arrived Muslim Arab Syrian refugees residing in Ontario	**n** = 10 **Age**: 30–55 years **Gender**: 50% females **Religion**: 100% Sunni Muslims **Education**: 70% elementary to high school; 30% college and university **Country of origin**: 100% Syria	Qual = 10/10 High
MakkiAlamdari (2020)	United States (HIC)	Quantitative: Cross-sectional study using questionnaire	War-affected Arab refugees in the US	**n** = 130 **Age**: ≥18 years (*M = 41, SD = 14, range = 18–72*) **Gender**: 40% females **Religion**: not specified **Education**: 5% no school but literate, 47% school, 19% some college, 29% university **Country of origin**: 77% Middle Eastern countries, 23% African countries, 2% other	Cross-sectional = 8/8 High
Nashwan et al. (2019)	United States (HIC)	Qualitative: Semi-structured interviews	Iraqi female refugees	**n** = 22 **Age**: 50–63 years (*M = 54.7, SD = 4.1*) **Gender**: 100% females **Religion**: 77.3% Muslims, 22.7% Christians **Education**: 9.1% none, 72.7% primary/secondary education, 18.2% post-secondary/undergraduate degree **Country of origin**: 100% Iraq	Qual = 9/10 High
Qureshi (2016)	United Kingdom (HIC)	Qualitative: Narrative inquiry and semi-structured interviews	Syrian male refugees	**n** = 3 **Age**: 21-35 years **Gender**: 0% females **Religion**: not specified **Education**: 67% university **Country of origin**: Syria	Qual = 10/10 High
Rayes et al. (2021)	Germany (HIC)	Qualitative: Semi-structured interviews	Arabic or Farsi speaking adults with refugee or asylum seeker status in Germany	**n** = 17 **Age**: 22-47 years (*M = 35*) **Gender**: 35% females **Religion**: 100% Muslims, 12% non-religious (or non-practicing) Muslims **Education**: not specified **Country of origin**: 71% Syria, 29% Iraq	Qual = 10/10 High
Renner et al. (2020)	Germany (HIC)	Qualitative: Semi-structured focus groups	Syrian refugees residing in Germany since 2015	**n** = 20 **Age**: ≥18 years (*M = 28, SD = 9, range = 20–60*) **Gender**: 20% females **Religion**: not specified **Education**: 30% university degree, 70% other **Country of origin**: 100% Syria	Qual = 8/10 High
Sim et al. (2023)	Canada (HIC)	Mixed Methods: Quantitative: (survey) & Qualitative (open ended questions)	Arabic-speaking refugee parents experiencing depression and anxiety living in Canada	**n** = 40 **Age**: ≥18 years (*M = 43.3, SD = 7.81*) **Gender**: 60% females **Religion**: not specified **Education**: 10% no formal education, 77.5% primary and secondary school, 12.5% post-secondary and above **Country of origin**: 82.5% Syria, 10% Iraq, 7.5% Sudan	Cross-sectional = 6/8 Medium Qual = 9/10 High
Tauson (2017)	Thailand (UMIC)	Qualitative: Participant observation & in-depth interviews	Palestinian-Syrian refugees in Bangkok	**n** = 9 **Age**: 20-50 years **Gender**: 22% females **Religion**: 100% Muslims **Education**: 11% tertiary education **Country of origin**: Palestine and Syria	Qual = 10/10 High
Zbidat et al. (2020)	Germany (HIC)	Qualitative: Semi-structured interviews	Syrian refugees residing in Germany since 2014 or less	**n** = 16 **Age**: ≥18 years (*M = 36, SD = 11, range = 21–55*) **Gender**: 50% females **Religion**: 81% Muslims, 13% Christians, 6% other **Education**: 43% school, 6% university degree, 18% other **Country of origin**: 100% Syria	Qual = 10/10 High
**Intervention Studies (n = 37)**					
Acarturk et al. (2022)	Turkey (UMIC)	Quantitative: randomised controlled trial (RCT)	Arabic-speaking Syrian refugees	***Intervention*** **n** = 322 **Age**: ≥18 years (*M = 31.2, SD = 9*) **Gender**: 64% females **Religion**: 42% Muslims **Education**: 5.28% illiterate, 62.73% primary, 16.46% high school, 14.60% university **Country of origin**: 98.4% Syria, 1.2% Iraq	RCT = 12/13 High
Ahmad et al. (2020)	Egypt (LMIC) and Germany (HIC)	Quantitative: Quasi-experimental (questionnaire)	Syrian refugee children from Egypt and Germany	**n** = 16 **Age**: ≥14 years (*M = 15, SD = 2, range = 14–18*) **Gender**: 25% females (both Egypt and Germany) **Religion**: not specified **Education**: not specified **Country of origin**: 100% Syria	Quasi-Experimental = 6/9 Medium
Aladdin & Hawamdeh (2021)	Jordan (UMIC)	Quantitative: RCT	Syrian refugee male students enrolled in the sixth and seventh grades	**n** = 40 **Age**: ≥12 years (*M = 13, SD = 1, range = 12–14*) **Gender**: 0% females **Religion**: not specified **Education**: school **Country of origin**: 100% Syria	RCT = 8/13 Medium
Al-Dmour & Al-Safasfeh (2020)	Jordan (UMIC)	Quantitative: RCT	Syrian refugee female students	**n** = 30 **Age**: 12-15 years **Gender**: 100% females **Religion**: not specified **Education**: school **Country of origin**: 100% Syria	RCT = 8/13 Medium
Al-Refai et al. (2022)	Jordan (UMIC)	Quantitative: RCT	Syrian refugees	**n** = 36 **Age**: 18–60 years **Gender**: 50% females **Religion**: 100% Muslims **Education**: school **Country of origin**: 100% Syria	RCT = 10/13 Medium
Al-Shahadat (2021)	Jordan (UMIC)	Quantitative: RCT	Syrian refugee female students enrolled in the 7th, 8th and 9th grade	**n** = 30 **Age**: 13-16 years **Gender**: 100% females **Religion**: not specified **Education**: school **Country of origin**: 100% Syria	RCT = 8/13 Medium
Alsheikh Ali (2020)	Jordan (UMIC)	Quantitative: Quasi-experimental (pre-posttest questionnaires)	Syrian refugee females in Jordan	**n** = 40 **Age**: 30-50 years **Gender**: 100% females **Religion**: not specified **Education**: not specified **Country of origin**: 100% Syria	Quasi-Experimental = 7/9 Medium
Blignault et al. (2019)	Australia (HIC)	Quantitative: Quasi-experimental (pre-post and follow-up questionnaire)	Arabic-speaking adults in Sydney	**n** = 70 **Age**: 18–65 years **Gender**: 73% females **Religion**: 94% Muslims **Education**: 49% no post-school qualifications **Country of origin**: 79% Lebanon, 21% other	Quasi-Experimental = 8/9 High
Blignault et al. (2021a)	Australia (HIC)	Mixed Methods: Quantitative Quasi-experimental (questionnaire) & Qualitative (participant feedback)	Arabic-speaking Muslim women in Sydney	**n** = 20 [PPA] **Age**: 18-65 years **Gender**: 100% females **Religion**: 100% Muslims **Education**: 45% post-school qualifications **Country of origin**: 55% Iraq, 20% Lebanon, 15% Syria, 10% Libya	Quasi-Experimental = 8/9 High Qual = 10/10 High
Blignault et al. (2021b)	Australia (HIC)	Mixed Methods: Quantitative (RCT) & Qualitative (participants’ log sheet comments)	Arabic-speaking community members in Sydney	**n** = 131 Arabic speakers [PPA] **Age**: 14% 16-25 years, 24% 26-35 years, 17% 36-45 years, 45% other (*M = 13*) **Gender**: 83% females **Religion**: 63% Muslims, 37% Christians, 1% none **Education**: 45% Year 12 or less, 39% university, 14% trade certificate **Country of origin**: 29% Lebanon, 21% Australia, 49% other	RCT = 9/13 Medium Qual = 10/10 High
Blignault et al. (2022)	Australia (HIC)	Mixed Methods: Quantitative (self-report questionnaires) and Qualitative (semi-structured interviews)	Arabic-speaking women in Australia	**n** = 18 **Age**: 26–55 years **Gender**: 100% females **Religion**: 80.76% Muslims, 11.5% Christians **Education**: 69% post-school qualification **Country of origin**: 92% born overseas (Iraq, Lebanon, Syria)	Quasi-experimental = 7/9 Medium Qual = 9/10 High
Bryant et al. (2022a) Bryant et al. (2022b)	Jordan (UMIC)	Quantitative: RCT	Arabic-speaking Syrian refugees living in Azraq Refugee Camp (Jordan)	***Intervention*** **n** = 204 **Age**: ≥18 years (*M = 39.38, SD = 6.71*) **Gender**: 71% females **Religion**: not specified **Education**: 27.5% none, 63.7% certificate, 6.8% secondary education, 2% university **Country of origin**: Syria	RCT = 11/13 High
Cuijpers et al. (2022)	Lebanon (UMIC)	Quantitative: RCT	Arabic-speaking Syrian displaced people/refugees in Lebanon	***Intervention*** **n** = 283 **Age**: ≥18 years (*M = 31.4, SD = 8.5*) **Gender**: 61.7% females **Religion**: not specified **Education**: 67.9% school, 26.1% university, 6% other **Country of origin**: 100% Syria	RCT = 12/13 High
de Graaff et al. (2020)	Netherlands (HIC)	Mixed Methods: Quantitative (RCT) and Qualitative (semi-structured interviews)	Adult Arabic-speaking Syrian refugees in Rotterdam, Netherlands	***Intervention*** **n** = 30 **Age**: ≥18 years (*M = 37.6, SD = 11.8*) **Gender**: 60% females **Religion**: not specified **Education**: 51.7% no or basic education, 24.1% secondary education and above **Country of origin**: Syria	RCT = 11/13 High Qual = 8/10 High
de Graaff et al. (2023)	Netherlands (HIC)	Quantitative: RCT	Arabic-speaking Syrian refugees, experiencing symptoms of PTSD, depression and anxiety living in the Netherlands	***Intervention*** **n** = 103 **Age**: ≥18 years (*M = 36.4, SD = 11.97, range = 18–69*) **Gender**: 29.1% females **Religion**: not specified **Education**: 9.7% no or basic education, 20.4% technical/vocational/associate degrees, 69.9% secondary to tertiary education **Country of origin**: Syria	RCT = 11/13 High
DePierro (2020)	Jordan (UMIC)	Quantitative: RCT	Syrian refugees, living in the Za’atri Refugee Camp in Jordan able to speak, read and write in Arabic	**n** = 128 **Age**: ≥18 years **Gender**: 49% females **Religion**: not specified **Education**: 100% literate **Country of origin**: Syria	RCT = 9/13 Medium
Doumit et al. (2020)	Lebanon (UMIC)	Quantitative: Quasi-experimental (questionnaires/surveys)	Adolescent Syrian refugees	**n** = 31 [PPA] **Age**: 13-17 years (*M = 14, SD = 1*) **Gender**: 52% females **Religion**: not specified **Education**: school **Country of origin**: 100% Syria	Quasi-Experimental = 8/9 High
Feen-Calligan et al. (2023)	United States (HIC)	Qualitative: Photovoice techniques	Arab American adolescents with refugee or immigrant background recently resettled in Michigan, US.	**n** = 8 **Age**: 15–17 years **Gender**: 100% females **Religion**: 100% Muslims **Education**: school **Countries of origin**: Syria, Palestine, Yemen, Lebanon, United Arab Emirates, and Iraq	Qual = 9/10 High
Gordon et al. (2016)	Gaza/Palestine (LMIC)	Quantitative: Quasi-experimental (questionnaire)	Palestinian adults in Gaza	**n** = 92 **Age**: 18–49 years (*M = 30*) **Gender**: 58% females **Religion**: not specified **Education**: 51% school, 49% postsecondary **Country of origin**: Palestine	Quasi-Experimental = 8/9 High
Hasha et al. (2022)	Norway (HIC)	Quantitative: RCT	Arabic-speaking adult Syrian refugees experiencing traumatic events	***Intervention*** **n** = 38 **Age**: ≥16 years (*M = 33, SD = 10.4*) **Gender**: 32% females **Religion**: not specified **Education**: years of education (*M = 10, SD = 4.8*) **Country of origin**: 100% Syria	RCT = 10/13 Medium
Husby et al. (2020)	Denmark (HIC)	Mixed Methods: Quantitative Quasi-experimental (questionnaire survey) & Qualitative (focus group interviews)	Arabic-speaking adult refugees from Syria and Palestine, living in Denmark and had obtained asylum no more than 5 years before the intervention	***Quantitative*** **n** = 92 (Quant) **Age**: ≥18 years **Gender**: 64% females **Religion**: not specified **Education**: not specified **Country of origin**: 41% Syria, 2% Palestine ***Qualitative*** In FGDs, n = 32	Quasi-Experimental = 6/9 Medium Qual = 8/10 High
Ibrahim (2017)	Sudan (LIC)	Quantitative: Quasi-experimental (questionnaire)	Syrian refugees residing in Khartoum state, Sudan	**n** = 20 **Age**: 20-60 years **Gender**: 55% females **Religion**: not specified **Education**: 90% primary, 10% high school **Country of origin**: 100% Syria	Quasi-Experimental = 7/9 Medium
Kayrouz et al. (2015)	Australia (HIC)	Quantitative: Quasi-experimental (pre, post and follow-up test using questionnaire)	People of Arabic ancestry living in Australia (Australia or overseas born)	**n** = 11 **Age**: 18-70 years (*M = 34, SD = 9, range = 25–50*) **Gender**: 73% females **Religion**: not specified **Education**: 73% Bachelor’s degree, 18% other **Country of origin**: Australia and overseas	Quasi-Experimental = 8/9 High
Kayrouz et al. (2016)	International (N/A)	Quantitative: Quasi-experimental (pre, post and follow-up test using questionnaire)	People of Arabic ancestry living in Australia and elsewhere	**n** = 36 **Age**: 18-70 years (*M = 36, SD = 12, range = 19–67*) **Gender**: 58% females **Religion**: not specified **Education**: 64% Bachelor’s degree, 36% other **Country of origin**: 53% Australia, 33% Middle Eastern countries (Lebanon, Egypt, Saudi Arabia), 5.5% UK, 5.5% USA, 3% Algeria	Quasi-Experimental = 8/9 High
Lindegaard et al. (2020)	Sweden (HIC)	Quantitative: Pilot RCT	Refugees and immigrants able to read and write Arabic fluently in Sweden	**n** = 59 **Age**: ≥18 years (*M = 38, SD = 11, range = 20–69*) **Gender**: 42% females **Religion**: not specified **Education**: 46% school, 46% completed or ongoing university education, 15% other **Country of origin**: not specified (78% refugee, 7% immigrant)	RCT = 8/13 Medium
Lindegaard et al. (2021)	Sweden (HIC)	Qualitative: Semi-structured interviews	Resettled refugees and immigrants, able to read and write Arabic residing in Sweden	**n** = 10 **Age**: ≥18 years (*M = 33, SD = 9, range = 20–49*) **Gender**: 60% females **Religion**: not specified **Education**: 40% school, 50% completed or ongoing university education, 10% other **Country of origin**: not specified (80% refugees, 10% immigrant)	Qual = 9/10 High
Mercy Corps (2020)	Jordan, Lebanon, and Iraq (UMIC)	Mixed Methods: Quantitative Quasi-experimental (survey) & Qualitative (KIIs and FGDs)	Adolescents and youths were a mix of Iraqi, Jordanian, Lebanese, Palestinian and Syrian, Arabs and Kurds, refugees, internally displaced people, and those from host communities	***Quantitative*** **n** = 1,607 **Age**: 12-19 years **Gender**: 41% females **Religion**: not specified **Education**: not specified **Country of origin**: 47% Lebanon, 39% Jordan 41% refugees, 45% from host communities ***Qualitative*** In KIIs n = 36; no precise gender breakdown for males and females In FGDs n = 32; 20 males, 12 females	Quasi-Experimental = 7/9 Medium Qual = 9/10 High
Nilsson et al. (2019)	Sweden (HIC)	Qualitative: FGDs	Arabic-speaking refugees, women and men	**n** = 33 **Age**: >18 years (*M = 45, range = 22–67*) **Gender**: 30% females **Religion**: not specified **Education**: not specified **Country of origin**: Iraq, Syria, Lebanon, Jordan, or Palestine	Qual = 10/10 High
Panter-Brick et al. (2018)	Jordan (UMIC)	Quantitative: RCT & Quasi-Experimental	Refugees (Syrian) and host-community (Jordanian) youth	**n** = 463 **Age**: 12-18 years (*M = 14, SD = 2*) **Gender**: 47% females **Religion**: not specified **Education**: highest educational level (*M = 7.04, SD = 2.15*) **Country of origin**: 54% Syria	RCT = 9/13 Medium Quasi-Experimental = 7/9 Medium
Powell et al. (2023)	Jordan (UMIC)	Qualitative: FGDs	Resettled Syrian refugees and Jordanians, experiencing symptoms of depression and impaired functioning	**n** = 21 **Age**: ≥18 years (*M = 48*) **Gender**: 66.7% females **Religion**: not specified **Education**: 14.3% no school, 61.9% secondary or high school, 19% some college **Country of origin**: 52% Jordan, 48% Syria	Qual = 10/10 High
Raknes (2020)	Lebanon (UMIC)	Mixed Methods: Quantitative (questionnaire) & Qualitative (open-ended questions, session reports and interviews)	Syrian displaced and refugee adolescents living in Lebanese informal settlements	**n** = 20 **Age**: 13-17 years (*M = 14*) **Gender**: 60% females **Religion**: not specified **Education**: 70% illiterate or nearly illiterate **Country of origin**: 100% Syria	Quasi-Experimental = 6/9 Medium Qual = 9/10 High
Rayes (2017)	Jordan (UMIC)	Quantitative: Quasi-experimental (pre-post study using survey)	Syrian refugee adolescents in 3 urban districts and 2 camp settings	**n** = 7,644 **Age**: 12-18 years **Gender**: 50% females **Religion**: not specified **Education**: not specified **Country of origin**: 100% Syria	Quasi-Experimental = 8/9 High
Röhr et al. (2021)	Germany (HIC)	Quantitative: Pilot RCT	Syrian refugees	***Intervention*** **n** = 65 **Age**: ≥18 years (*M = 33, SD = 11, range 18–65*) **Gender**: 34% females **Religion**: not specified **Education**: 42% high education level, 32% medium, 26% low **Country of origin**: 100% Syria	RCT = 13/13 High
Skarneh & Ghaith (2020)	Jordan (UMIC)	Quantitative: RCT	Syrian refugee women	**n** = 30 **Age**: 18-50 years **Gender**: 100% females **Religion**: not specified **Education**: not specified **Country of origin**: 100% Syria	RCT = 11/13 High
Slewa-Younan et al. (2020)	Australia (HIC)	Quantitative: Quasi-experimental (pre, post and follow-up using questionnaire)	Arabic-speaking women and men	**n** = 33 **Age**: ≥18 years (*M = 48, SD = 9*) **Gender**: 58% females **Religion**: not specified **Education**: years of education *(M = 8.8, SD = 3.7)* **Country of origin**: Syria and Iraq	Quasi-Experimental = 8/9 High
Spaaij et al. (2022)	Jordan (UMIC)	Mixed Methods: Quantitative (RCT) and Qualitative (semi-structured interviews)	Arabic-speaking Syrian refugees and asylum seekers	***Quantitative*** ***Intervention*** **n** = 31 **Age**: ≥18 years (*M = 39.5, SD = 10.67*) **Gender**: 45.2% females **Religion**: not specified **Education**: 80.6% basic & secondary, 19.4% university **Country of origin**: Syria ***Qualitative*** **n** = 18 **Age**: ≥18 years (M = 39.4, *SD* = 9.33) **Gender**: 50% females	RCT = 10/13 Medium Qual = 9/10 High
Tashtoush & Khawaldeh (2020)	Jordan (UMIC)	Quantitative: RCT	Syrian refugee students	**n** = 30 **Age**: 13-15 years (estimated) **Gender**: not specified **Religion**: not specified **Education**: school **Country of origin**: 100% Syria	RCT = 9/13 Medium

### Participant characteristics

#### Observational studies

Of the observational studies (sample size from 3 to 6,375), sixteen focused solely on Arabic-speaking refugees [50, 51, 54, 55, 60–65, 67–70, 88, 89], two on refugees and the host community [53, 90], one on refugees and migrants [66], and one solely on migrants [48, 49]. Countries of origin were mainly Palestine, Syria and Iraq. The mostly female participants ranged in age from 14 to over 80 years, with most being youths and young adults. Nineteen studies reported on participants’ literacy or education; most were literate [48–51, 53–55, 60–70, 89, 90]. Thirteen studies reported participants’ religion; most were practicing Muslims [48–51, 55, 60, 62, 64–66, 68, 69, 89, 90].

#### Intervention studies

The sample size for the intervention studies ranged from 8 to 7,644. Twenty-six studies focused on refugees or those with a refugee-like background [57, 58, 71, 73–76, 78–86, 91–101], three on refugees and the host community [72, 77, 87], three on refugees and migrants [102–104], and five solely on migrants [105–109]. Lebanon, Syria, Jordan and Palestine were the main countries of origin. As in the observational studies, most participants were female. They ranged in age from 12 to 70 years, with most aged 12–18 years. Twenty-nine studies reported on participants’ education; most were university educated [57, 58, 72, 73, 75, 76, 78, 80–82, 84–87, 91, 92, 96–109]. Eight studies reported participants’ religion; most were Muslims [83, 84, 98, 99, 104, 107–109].

### Mental health self-care strategies

After reviewing all included studies, we found mental health self-care activities fell into four broad groups—social strategies, psychological strategies, religious/spiritual strategies, and other strategies; findings were consistent with research on self-care and coping in other populations including African migrants and East African refugees [110–113]. Social strategies involved connecting with social networks. Psychological strategies were of two types—cognitive and behavioural. Cognitive strategies involved relying on inner resources to positively reframe negative situations. Behavioural strategies included taking action to address daily life stressors. Religious/spiritual strategies included relying on faith to find meaning in life and performing religious activities. The final group of strategies comprised various activities related to keeping oneself busy, including engaging in physical activity, pursuing hobbies or studying.

Table 2 summarises the broad self-care strategies reported in the included studies, and the study outcome measures where applicable. After this, we detail the different strategies included in each of the four groups, with the findings organised by study type.

**Table 2:** Self-care strategies and study outcome measures by study type

Author (Year)	Self-care strategy				Outcome measures
	Social	Psychological	Religious/Spiritual	Other	
**Observational Studies (n = 20)**					
Al-Ajarma (2010)	✓	✓	✓	✓	NA
Alhaddad et al. (2021)	✓	✓	✓	✓	NA
Boswall & Akash (2015)	✓		✓	✓	NA
Chaaya et al. (2007)			✓		GDS-15
Darychuk & Jackson (2015)	✓		✓	✓	NA
Ferguson (2015)	✓	✓	✓	✓	NA
International Medical Corps (2017)	✓	✓	✓	✓	NA
International Medical Corps Lebanon (2011)	✓	✓	✓	✓	NA
Irfaeya (2006) Irfaeya et al. (2008)	✓	✓	✓	✓	NA
Jamil (2020)	✓	✓	✓	✓	NA
Kadri (2009)	✓	✓	✓		NA
Keshavarzi (2018)	✓		✓	✓	NA
Makki Alamdari (2020)	✓	✓	✓	✓	NA
Nashwan et al. (2019)	✓	✓	✓	✓	NA
Qureshi (2016)	✓	✓	✓	✓	NA
Rayes et al. (2021)	✓		✓		NA
Renner et al. (2020)	✓	✓	✓	✓	NA
Sim et al. (2023)	✓	✓	✓	✓	NA
Tauson (2017)	✓	✓	✓	✓	NA
Zbidat et al. (2020)	✓	✓	✓	✓	NA
**Intervention Studies (n = 37)**					
Acarturk et al. (2022)	✓	✓			MINI, GHQ-12, PCL-5, PHQ-9, PSYCHLOPS, WHO- DAS 2.0, WHO-5, EQ-5D-3 L
Ahmad et al. (2020)	✓	✓	✓	✓	PCL-5
Aladdin & Hawamdeh (2021)		✓		✓	SDQ
Al-Dmour & Al-Safasfeh (2020) *		✓			PTSD scale Satisfaction with Life scale
Al-Refai et al. (2022) *	✓	✓			Psychological Empowerment Scale
Al-Shahadat (2021) *		✓			Psychological stress scale Self-esteem scale
Alsheikh Ali (2020)		✓			PCL, PWB
Blignault et al. (2019)		✓	✓		K-10+, DASS-21
Blignault et al. (2021a)	✓	✓	✓		DASS-21
Blignault et al. (2021b)	✓	✓	✓		K-10+, DASS-21
Blignault et al. (2022)	✓	✓	✓		K-10+
Bryant et al. (2022a) Bryant et al. (2022b)		✓			HSCL-25, PCL-5
Cuijpers et al. (2022)		✓			PHQ-9, WHODAS-12 2.0, WHO5WBI, GAD-7, PCL-5, PSYCHLOPS
de Graaff et al. (2020)	✓	✓			HSCL-25, WHODAS 2.0, PCL-5, PSYCHLOPS
de Graaff et al. (2023)		✓			HSCL-25, WHODAS 2.0, PCL-5, PSYCHLOPS
DePierro (2020)	✓	✓		✓	ITQ, K-10
Doumit et al. (2020)	✓	✓			PHQ-9, GAD-7, PedsQL
Feen-Calligan et al. (2023)	✓	✓	✓	✓	Self-report from selected participants
Gordon et al. (2016)	✓	✓		✓	HTQ, HSCL-25, WHOQOL-BREF
Hasha et al. (2022)		✓			IES-R, GHQ-12
Husby et al. (2020)	✓	✓			WHO-5
Ibrahim (2017)		✓	✓		DTS
Kayrouz et al. (2015)		✓			PHQ-9, GAD-7, K-10
Kayrouz et al. (2016)		✓	✓		PHQ-9, GAD-7, K-10
Lindegaard et al. (2020)		✓			PHQ-9, IES-R, GAD-7
Lindegaard et al. (2021)		✓			Self-report from selected participants
Mercy Corps (2020) *	✓	✓		✓	AYMH
Nilsson et al. (2019)	✓	✓		✓	Self-report from selected participants
Panter-Brick et al. (2018) *	✓	✓		✓	AYMH, CRIES-8
Powell et al. (2023)	✓	✓	✓	✓	NA
Raknes (2020) *		✓			WHO5WBI
Rayes (2017) *		✓		✓	YEP index
Röhr et al. (2021)		✓			PDS-5, PHQ-9, GAD-7
Skarneh & Ghaith (2020)	✓	✓		✓	HTQ
Slewa-Younan et al. (2020)		✓		✓	K-10
Spaaij et al. (2022)		✓			HSCL-25, PCL-5
Tashtoush & Khawaldeh (2020) *	✓	✓			Psychological and social adjustment scale

Translated Arabic versions of the psychometric scales were used

*These scales were developed in Arabic

AYMH, Arab Youth Mental Health; CRIES-8, Child Revised Impact of Events Scale; DASS-21, Depression Anxiety and Stress Scale; DTS, Davidson Trauma Scale; EQ-5D-3 L, European Quality of Life 5-Dimensions 3-Level; GAD-7, Generalized Anxiety Disorder; GHQ-12, General Health Questionnaire; GDS, Geriatric Depression Scale; HSCL-25, Hopkins Symptom Checklist-25; HTQ, Harvard Trauma Questionnaire; IES-R, Impact of Event Scale-Revised; ITQ, International Trauma Questionnaire; K-10, Kessler Psychological Distress Scale; MINI, Mini International Neuropsychiatric Interview; PCL-5, PTSD Checklist; PDS-5, Posttraumatic Diagnostic Scale; PedsQL, Paediatric Quality of Life Inventory; PHQ-9, Patient Health Questionnaire; PSYCHLOPS, Psychological Outcome Profiles; PWB, Ryff’s Psychological Well-Being; SDQ, Strengths and Difficulties Questionnaire; WHO-DAS 2.0, WHO Disability Assessment Schedule 2.0; WHO-5/WHO5WBI, World Health Organization-Five Well-Being Index; WHOQOL-BREF, World Health Organization Quality of Life Instrument; YEP, Youth Empowerment Program Index

### Observational studies

#### Social strategies

Nineteen observational studies reported the use of social strategies. This involved having a network of immediate and extended family, friends, and neighbours and connecting with them in person and via phone or social media [48–50, 55, 60, 62, 64, 65, 67, 88]. Talking with family, community members, authority figures, social service providers, and those with similar experiences for emotional support, advice, assistance and resources was common [53, 61, 63, 65, 66, 68, 70]. Raising children together, as a community with traditional values; participating in informal women’s groups where they could bring their children and discuss problems; and organising or attending cultural events to connect with new people of the same culture, faith or ethnicity were also reported [51, 53–55, 69, 88, 89].

#### Psychological strategies

Fifteen studies reported psychological strategies. Cognitive strategies involved positive thinking and positive self-talk, thinking deeply, changing mindset, realism, goal-setting, and being future-focused [48, 49, 54, 55, 60, 62, 63, 66–68, 70, 89]. Behavioural strategies involved active problem-focused strategies such as talking about problems and taking steps to improve situations, writing about stressors, and distraction with other activities [48, 49, 53, 54, 62, 66, 68, 70, 89]. Mind-body techniques such as slow breathing and meditation were noted [50, 61, 68]. Studies in high-income resettlement countries (Germany, New Zealand, United States and United Kingdom) and transit (Thailand) countries highlighted building self-determination, self-motivation, self-efficacy, self-control, perseverance, persistence and willpower to overcome obstacles [55, 60, 62–65, 68, 89].

#### Religious/spiritual strategies

All twenty studies reported strategies related to spirituality or religion (Islam and Christianity). These included praying and offering supplications, fasting, attending religious talks and services at churches or mosques (Friday prayers), meeting with others of the same faith, seeking help from local religious leaders, reading and listening to religious scriptures, and using Arabic expressions such as ‘Thank God’ and ‘God willing’ to convey hope for a better future [48–51, 55, 60, 61, 63, 64, 68, 69, 88–90]. Spirituality, having faith in a higher power, accepting and surrendering to God’s will, believing in divine judgement based on the consequences of one’s actions, and expressing gratitude to God were also mentioned [50, 51, 53–55, 61–67, 70, 88, 89].

#### Other strategies

Seventeen studies identified other strategies—leisure activities, daily life activities, expressive arts, physical activity and self-development. Leisure activities included watching television and comedy programs, going to the cinema, watching humorous videos and playing games on mobile phones, vacations, and reading [48–51, 54, 55, 61–64]. Seeking pleasure from daily life involved partaking of sustenance (food and sleep), doing housework, gardening, and spending time with children [48–50, 54, 55, 62, 64, 68]. Expressive arts practice included poetry, music, dance, singing, drawing, journaling, acting, filmmaking and photography [48–50, 55, 61–63, 67, 68]. Physical activities included sports, exercise walks, and going to the gym [48–50, 55, 62, 63, 66–68]. Studies, particularly in resettlement countries, reported self-development through learning the host country language, education, employment, and volunteering [48, 49, 51, 53, 55, 61–65, 67, 70, 88, 89].

### Intervention studies

As detailed in Table 3, 25 of the 37 interventions were delivered in groups [57, 58, 71–74, 76–84, 87, 91, 93–96, 98, 100, 104, 108, 109], 11 were delivered individually [85, 86, 92, 97, 99, 101–103, 105–107], and one was delivered in both formats [75]. Most interventions were delivered in person [57, 58, 71–74, 76, 77, 79–84, 86, 87, 91–98, 100, 108], with four also having a digital component [75, 78, 101, 104]. Seven interventions were delivered online [85, 99, 102, 103, 105, 106, 109] and one via compact disc [107]. Interventions were predominantly delivered in community settings [57, 58, 71–78, 80–85, 93–99, 101–109]. Twenty-two of the interventions were delivered by mental health professionals [71, 73, 74, 76, 78–82, 84, 91, 92, 94, 96, 98, 100, 102, 103, 105, 107–109], while ten were delivered by trained peer volunteers [57, 58, 72, 75, 77, 83, 85, 86, 95, 97, 101].

**Table 3:** Intervention details

Author (Year)	Intervention			Setting	Provider training/skill
	Duration	Format	Delivery		
Acarturk et al. (2022)	5 weeks; 5 sessions each 2 h	Group	In-person	Refugee camps/city settlements	Trained peer non-specialist facilitators
Ahmad et al. (2020)	4 weeks; 15 sessions each 1.5 h	Group	In-person	City settlement	Mental health and education researchers
Aladdin & Hawamdeh (2021)	14 weeks; 14 sessions each 45–50 min	Group	In-person	Elementary school hall	Skilled in CBT counselling
Al-Dmour & Al-Safasfeh (2020)	7 weeks; 14 sessions, twice/week and each for 60 min	Group	In-person	School	Educational psychologist, special education researchers, counsellors
Al-Refai et al. (2022)	12 weeks; 12 sessions, over 3 months, with one session a week, each for 60 min	Group	In-person	City settlement	Researchers with Islamic studies, Psychology and ICT background
Al-Shahadat (2021)	7 weeks; 14 sessions; two sessions/week, each 45–60 min	Group	In-person	School theatre hall	Family counselling skills
Alsheikh Ali (2020)	7 weeks; 2 sessions/week over 1.5 months	Group	In-person	City settlement	Licensed psychotherapist/clinical psychologist
Blignault et al. (2019)	5 weeks; Instructions to listen to 2–3 tracks at least 3 times a week over 4 weeks, and one track 3 times a week in the 5th week. 60 min/week	Individual	Compact disc	Home	Psychologist
Blignault et al. (2021a)	5 weeks; one session per week over five weeks	Group	In-person	NGO; Home	Psychologist supported by a bilingual multicultural health worker
Blignault et al. (2021b)	5 weeks; once a week over 5 weeks, most groups held in the morning and a couple in the evening	Group	In-person	Migrant resource centres and community facilities attached to mosques and churches; Home.	Psychologists and trained community workers
Blignault et al. (2022)	4 weeks; session once a week for four weeks	Group	Online	Home	Bilingual (Arabic/English) mental health clinician (psychologist) and bilingual community worker
Bryant et al. (2022a) Bryant et al. (2022b)	5 weeks; 5 sessions, 2 h	Group separated by gender	In-person	Refugee camp	Trained facilitators, proficient in Arabic
Cuijpers et al. (2022)	5–8 weeks; 5 sessions, 20 min (average)	Individual	Online	Home	Trained non-specialists (e-helpers) with psychology or health background. User contact via phone or message
de Graaff et al. (2020)	5weeks; 5 weekly sessions, each 90 min	Individual	In-person	Local NGO	Trained Arabic-speaking Syrian non-specialist peer-refugee helpers
de Graaff et al. (2023)	5 weeks; 5 sessions, 90 min	Individual	In-person and online	University or home	Trained Arabic-speaking Syrian non-specialist peer-refugee helpers
DePierro (2020)	Workshop condition: 8 weeks; 16 sessions 120 min twice weekly Reading at Home condition: weekly program content review, twice weekly SMS text reminders	Workshop condition: Group Reading at Home condition: Individual	In-person and SMS	Refugee camp	Trained community facilitators, with personal experiences as refugees and life in refugee camps
Doumit et al. (2020)	7 weeks; one weekly session each 60 min	Group	In-person	Community centre	Licensed instructor and therapist
Feen-Calligan et al. (2023)	10 weeks; 10 sessions for 5 months	Group	In-person and online	Museum; Home	Photography instructor, art therapy student interns, and their faculty supervisor, with support from the museum staff. Artistic and technical guidance via WhatsApp or Zoom
Gordon et al. (2016)	10 weeks; 10 sessions/week of 2 h	Group	In-person	Health clinic	Mental health professionals and educators supervised by Centre for Mind-Body Medicine (CMBM) faculty
Hasha et al. (2022)	6 weeks; 2.5 h weekly group sessions	Group separated by gender	In-person	The Centre for Crisis Psychology, University of Bergen	Research team members with prior experience working with refugees
Husby et al. (2020)	[Weeks undetermined]; 9 sessions of 2 h each	Group	In-person	Local (Danish) municipalities	Trainer of refugee background, social worker as co-trainer and an interpreter
Ibrahim (2017)	3 weeks; 6 sessions (2 sessions/week), each 45–60 min over 2 months	Individual	In-person	Not specified	Psychologist
Kayrouz et al. (2015)	8 weeks; 5 sessions	Individual	Online	Home	Bilingual counsellor and therapist Clinical support via email and phone
Kayrouz et al. (2016)	8 weeks; 5 sessions	Individual	Online	Home	Automated weekly emails. No direct contact or clinical support
Lindegaard et al. (2020)	8 weeks	Individual	Online	Home	Therapist support via email
Lindegaard et al. (2021)	8 weeks	Individual	Online	Home	Therapist support via email
Mercy Corps (2020)	13 weeks (approx.) for 3 months	Groups separated by gender	In-person	Community centres	Local volunteers, coaches and life-skill trainers with refugee background
Nilsson et al. (2019)	[Weeks undetermined]; 1–2 h sessions	Groups separated by gender	In-person	NGO	Physiotherapists
Panter-Brick et al. (2018)	8 weeks; 16 sessions usually 2 sessions/week	Groups separated by gender and age	In-person	Youth centres	Lay volunteers/trained local coaches
Powell et al. (2023)	[Weeks undetermined]; 4 sessions over a year, each 45 min (approx.)	Group	In-person	Primary care clinic	Health educators and nurses from local health clinics
Raknes (2020)	10 weeks; 10 sessions weekly	Group	In-person (smartphone/tablet-based game app)	Refugee camp tents	Psychologist (displaced Syrian background)
Rayes (2017)	10 weeks	Group	In-person	Urban refugee camps	Mental Health and Psychosocial Support (MHPSS) team
Röhr et al. (2021)	4 weeks	Individual	Online (smartphone-based app)	Home	NA
Skarneh & Ghaith (2020)	8 weeks; 15 sessions over 2 months, each 90–120 min, twice per week	Group	In-person	Healthcare institute	Counsellors from the local community
Slewa-Younan et al. (2020)	4 weeks; 3 h sessions weekly	Group	In-person	TAFE College, Adult Migrant English Program	Mental health clinicians and/or bilingual health educators
Spaaij et al. (2022)	5 weeks; Five 90-minute sessions once a week	Individual	In-person	Outpatient clinic	Trained Syrian non-specialist ‘helpers’ fluent in Arabic and German or English.
Tashtoush and Khawaldeh (2020)	7 weeks; 14 sessions, twice per week for 90 min each	Group	In-person	School	Counsellor or educational psychologist familiar with the local community and culture

### Intervention studies

#### Social strategies

Eighteen of the 25 group interventions clearly promoted social strategies—engaging with peers having similar problems, empathising with others and motivating them, providing a safe space for dialogue and opportunities to build connections, and continuing to meet for support post-intervention [72, 75–77, 79, 80, 83, 84, 87, 91, 93–95, 97, 98, 104, 108, 109]. Trained facilitators played key roles in building social cohesion, rapport and trust, leading to participants openly disclosing their problems in both group and individual formats [72, 77, 83, 93, 97, 98, 104].

#### Psychological strategies

All 37 interventions incorporated psychological strategies. In the 25 group interventions, these were typically based on cognitive behavioural therapy (CBT), cognitive behavioural play therapy (CBPT), narrative therapy, teaching recovery techniques (TRT), and emotional or behavioural self-regulation [71–74, 76–78, 80–83, 93–95, 100]. Cognitive restructuring and problem-solving techniques were often reported [57, 58, 71, 73, 74, 76, 78, 80–82, 84, 87, 93–95, 104]. Mind-body techniques included deep-breathing, yoga, mindfulness and meditation [73, 76, 79, 86, 87, 91, 93, 95–98, 101, 108, 109]. The 11 individual interventions included in-person, online and smartphone app-based CBT-informed techniques [85, 86, 92, 97, 99, 101–103, 105–107]. One hybrid intervention included psychoeducation, mindfulness, and self-care exercises delivered in groups and individually [75].

#### Religious/spiritual strategies

Six group interventions incorporated a religious or spiritual component, namely, designing CBT and mindfulness program content according to participants’ spiritual and religious beliefs, writing positive religious statements as daily practice, or praying [87, 94, 98, 104, 108, 109]. Three individual interventions, involving in-person and online CBT and compact disc-based mindfulness therapy respectively, were similarly tailored to participants’ faith and culture [92, 106, 107].

#### Other strategies

Twelve interventions incorporated other strategies, with four involving physical activity—exercise, nature walks, football, and a physiotherapeutic intervention (tension and trauma releasing exercises and aerobics) [72, 77, 87, 93]. Eight interventions involved expressive arts—drawing, craftwork, graphic design, photography, music, storytelling, theatre and drama [72, 75, 77, 79, 82, 91, 94, 104]. Skill-based psychosocial programs focused on vocational skills and mental health promotion and literacy [71, 72, 77, 91, 96].

### Effectiveness

Thirty intervention studies incorporating CBT, mind-body techniques or psychosocial programs showed statistically significant improvements in psychological wellbeing, quality of life, life satisfaction, and social cohesion, with a reduction in symptoms of psychological distress, depression, generalised anxiety and PTSD on a range of psychometric scales as listed in Table 2 [57, 58, 71–86, 91, 92, 94–98, 101, 105–109]. Fourteen studies reported improved outcomes maintained at follow-up [57, 58, 79, 81–83, 85, 86, 91, 94, 96, 97, 101, 105, 107]. Four studies reported a reduction in depression symptoms but not for anxiety or PTSD [90, 100, 102, 103]. Two studies noted self-reported reductions in somatic symptoms of depression, anxiety and PTSD [93, 104]. Only one intervention, the smartphone based *Sanadak* app, reported no statistically or clinically significant outcomes for refugees as compared to the control group. The authors suggest that the app may be more effective with support from trained facilitators [99].

### Cultural appropriateness

Twenty-seven intervention studies provided information relating to cultural appropriateness. Interventions were delivered in Arabic, with culturally and linguistically appropriate program content, led by non-specialist instructors with similar culture or refugee background, same language, and gender as the participants, in safe gender-segregated groups [57, 58, 72, 75–78, 82, 83, 85–87, 93, 95, 97–101, 103, 104, 107, 108]. The self-care components were grounded in participants’ sociocultural and religious practices and were beneficial and easy-to-use independently. Provision of refreshments during group interventions and eating together reflected cultural norms [76, 108]. Eight studies reported high program attendance and compliance and completion rates [75, 76, 98, 104, 106–109], which may be taken as an indication of broad cultural acceptability. Popularity of the strategies spread by word-of-mouth to family nearby and overseas, friends, and community members including those with mental disorders [75, 78, 87, 91, 98, 105, 106, 108, 109].

### Participant experiences

#### Social strategies

Twelve observational studies reported that activities involving family and friends were the most common source of emotional and practical support among Arabic-speaking refugees and migrants, with participants feeling safe, resilient, motivated, happy, and experiencing a sense of community, ease and belonging [48, 49, 53–55, 60–64, 66, 67, 70]. The following quotes are illustrative.

*… We talk about our kids and we cook. We share all the food we make together throughout the community. We most often talk about life in general. Like the things that stress us. We talk about how we want to make ourselves happy. We talk also about depression and mental health.*

Palestinian female refugee (age unspecified), West Bank, Palestine [88, p.452].

*The only feeling of safety I felt was when I was with my family.*

28-year-old Palestinian female refugee, United States [61, p.88].

Participants in thirteen intervention studies reported becoming more communicative and expressive about their feelings, which improved their relationships with family and friends [72, 75–77, 79, 80, 87, 93, 95, 97, 98, 104, 108].

*[Hearing] the calamity of others makes your calamity easier for you.*

Syrian female refugee (age unspecified), Jordan [79, p.288].

*I considered her [peer facilitator] a friend. Not a durable friendship but friendship in the session’s time. I was feeling comfortable talking to her.*

Syrian refugee (age and gender unspecified), Netherlands [97, p.19].

Key challenges included feelings of isolation and distress due to inadequate emotional and practical support from settlement workers, triggering of past trauma by peers, and limited opportunities for outdoor socialisation, particularly for female migrants and refugees [48, 49, 70, 88, 95].

#### Psychological strategies

Nine observational studies reported participants becoming more ambitious, determined, adaptable, resourceful and self-reliant [55, 60, 62, 63, 65, 66, 68, 70, 89].

*I always try to convince myself that I am stronger than the situation and can survive it.*

Syrian refugee (age and gender unspecified), Germany [55, p.9].

*It [hard times before coming to Thailand] is behind us; why do I want to think about these things? Sure I was scared, but now I am not, what is the point?*

Palestinian-Syrian female refugee in her 40s, Thailand [89, p.183].

Eleven of the intervention studies noted that participants practicing mind-body techniques and relaxation exercises reported feeling calmer, energised and mindful [75, 76, 79, 86, 87, 93, 95, 97, 98, 103, 107, 108]. They reported functioning better at school and work, and within the family [76, 86, 93]. Through learning about mental health awareness and ways to cope with psychological distress, participants became more compassionate, confident and empowered [72, 76–78, 80, 93–95, 98, 103, 108]. Six studies reported improved sleep; reduced feelings of anxiety and depression; lower intake of medications; higher referrals to psychologists and overall improved quality of life and wellbeing [77, 93, 98, 103, 108, 109].

*Sometimes I remembered my children they are away…so I breath in and out and feel relaxed.*

Syrian male refugee (age unspecified), Jordan [87, p.167].

*The way I integrate myself is for an example by active listening, which we have discussed. It is to listen to your children. […] Things like this make me better mentally and psychically. This makes me able to take time to be a part of society.*

Female refugee (age unspecified), Denmark [95, p.1035].

Ten studies reported psychological interventions (group or individual) with a digital component [78, 85, 99, 101–106, 109]. Using psychological self-care strategies via internet and smartphone apps, participants felt supported by facilitators and found them convenient due to privacy and time and cost efficiency [78, 85, 99, 103, 105, 109]. However, participants also faced some challenges while practicing self-care strategies adopted from the interventions due to stigma against mental illness, work commitments; technological problems (e.g., lack of electronic devices, online platform access, internet and electricity); low literacy; difficulty understanding, retaining and applying the program concepts; dissatisfaction with program content and short duration; inability to practice self-care exercises due to insufficient focus or physical pain and emotional distress; trauma triggers; distractions at home; and negative interaction with facilitators [78, 86, 95, 97, 98, 103, 108].

#### Religious/spiritual strategies

Eighteen of the observational studies stated that practicing religious and spiritual strategies during flight and afterwards served as sources of comfort, security, hope, and peace [48, 49, 51, 53–55, 60, 62–66, 68–70, 88–90].

*It [praying or listening to Quran recitations] makes you feel better and comforts all your pains.*

Syrian female refugee (age unspecified), Jordan [51, p.211].

Five of the intervention studies highlighted the benefits of daily prayers and religious practices [87, 98, 104, 107, 108].

*Most important point for me is that mindfulness relates to my religion. For example, prayers require the person to be mindful. My mind used to wander during prayers. Mindfulness has strengthened my faith and reduced stress*.

Muslim female refugee (age unspecified), Australia [98, p.9].

Male refugees in Germany expressed concerns about the lack of nearby mosques, especially those of the same Islamic sect and language, praying less frequently than in their country of origin, missing Friday prayers due to work, and distrust of and lack of engagement with imams (religious clerics) [69].

*I am finding some difficulties in maintaining prayer here. In Syria, I used to never miss a prayer, but not because I am less convinced [by my faith]. It is a shortage on my end. Near my house, there is no mosque near my house. The closest one is an hour away. My faith practice is inside my house, mainly.*

Syrian Muslim male refugee (age unspecified), Germany [69, p.6].

#### Other strategies

Participants in sixteen observational studies reported feeling inspired and confident by keeping busy with various self-development activities and hobbies without facing any overt challenges [48–51, 54, 55, 61–68, 70, 88, 89].

*For me, music was a relief. I would feel relaxed even when I blew into the flute without actually playing it or when holding the flute in my hand. It had become an inseparable companion.*

35-year-old female with refugee background, United States [61, p.109].

Eight interventions incorporating other self-care strategies, particularly expressive arts and physical activity, reported participants feeling relaxed and forgetting previous trauma and daily stressors [72, 75, 77, 79, 87, 93, 96, 104]. For example,

*The sports make us forget. I think about winning, and I laugh with my friends. … There is nothing that can make us forget what has happened to us, or make our problems go away, but at least we have this.*

37-year-old male refugee, Sweden [93, p.7].

*… I use photography as a way of relaxation at stressful times.*

Adolescent female with immigrant-refugee background, United States [104, p.8].

## Discussion

This systematic review sought to identify and examine the global evidence on mental health self-care strategies for Arabic-speaking refugees and migrants, addressing depression, anxiety and PTSD, which are prevalent among these populations [6–10, 114, 115]. Over the past decade, this area has attracted increased attention from researchers prompted by political unrest and violent conflict in the Middle East and the resultant rise in Arabic-speaking refugees and asylum seekers. Since 2014, Syria has been a major source country for refugees, with approximately 13.5 million forcibly displaced people worldwide [116]. A comprehensive search of the published and grey literature in English and Arabic since 2000, identified 37 interventions and 20 observational studies that met the inclusion criteria. The majority of studies were dated from 2020 onwards, conducted in upper-middle and high-income countries, and focused on refugees. Across both study types, four broad groups of mental health self-care strategies were identified: social, psychological, religious/spiritual and other (including expressive arts and physical activity). Psychological strategies featured in all the interventions. Social and religious/spiritual strategies were more commonly reported in observational studies, although psychological strategies were also utilised. Interventions that integrated self-care components based on cultural and religious practices of the target community resulted in positive user experiences and improved mental health and wellbeing.

Contextual factors are critical in designing and implementing local solutions for global health concerns, such as mental health interventions for refugees and asylum seekers [117]. This review suggests that there are many similarities in self-care strategies used by Arabic-speaking refugees in transit and resettlement countries, including finding comfort in religious and spiritual beliefs, seeking support from social connections, and engaging in relief-providing activities. An Australian qualitative study with 23 Sudanese refugees (mostly Christians with 10 Arabic speakers) found that while they experienced various difficulties across pre-migration, transit and post-migration phases, the self-care strategies they employed were similar; relying mainly on religion, social networks and cognitive approaches [110].

### Social strategies

Social strategies identified in this review, such as maintaining a strong network of family, friends and community, elicited feelings of safety, belonging and resilience. Research highlights the mental health benefits of social connections and receiving emotional or practical support from them, with limited peer connections linked to overall poor mental health and increased depression symptoms [118–121]. Digital technology was commonly reported in both observational and intervention studies. Four studies in our review [50, 51, 65, 66] noted that refugees and migrants used social media and phone calls to family and friends in the home country, supporting the role of technology for social connection [122]. Group interventions with self-care components, facilitated by trained peers, often involved social support. This aligns with a recent systematic review on mental health intervention delivery amongst refugees and asylum seekers in low-and middle-income countries (LMICs), highlighting the role of trained facilitators, usually well-respected community members, in guiding participants and fostering strong relationships to improve program feasibility [117].

Within Middle Eastern Arab cultures, Muslims and Christians pursue happiness and satisfaction through mutual assistance and fulfilment of social duties [123–127]. Seeking support from family, friends and community is deeply rooted in the collectivist culture and identity [33, 128–130]. Following traditional customs and bonding over meals shared with family and community help in building trust, agency and resilience among Arabic-speaking refugees [130–132]. We identified one study of Palestinian women in West Bank refugee camps where raising children within the community and upholding traditional values contributed to enhanced resilience and mental wellbeing [88]. The cultural significance of sharing food and communal eating was noted in two studies [76, 108].

Two studies reported significant challenges related to gender, such as inadequate female-friendly recreation facilities and unsafe meeting spaces, particularly, but not only, in refugee camps [48, 49, 88]. Unsafe living conditions were associated with depression and PTSD symptoms, especially among women [133–136]. Generally, women experience higher rates of mental disorders than men [137, 138]. Arabic-speaking women often face limitations on public interactions and need to be accompanied by a male family member when outside [139–141]. Traditional beliefs also restrict women’s participation in physical recreation [142, 143].

### Psychological strategies

Cognitive self-care generally involved utilising internal resources such as inner strength, positive self-talk and optimism. Interventions with self-care components often introduced CBT skills such as problem-solving, cognitive restructuring and mind-body techniques. Participants who practiced these psychological strategies reported an improved quality of life and mental wellbeing. Mind-body techniques are popular, easy-to-use, affordable and clinically effective in reducing anxiety, depression, and PTSD symptoms, and can be considered in psychological interventions for migrants, refugees and asylum seekers [144–146].

Challenges in practicing psychological strategies, including limited device access and low literacy levels were identified in seven studies. While these challenges are common among migrants and refugees [147–149], culturally tailored technology-mediated platforms, including self-care apps, show promise in transit settings (UMICs and LMICs) with limited mental health services [150, 151]. However, it is recommended to supplement app-based platforms with on-demand guidance from health professionals sharing the same race, gender, religion or language as the Arabic-speaking migrants and refugees [149, 152–155].

Group interventions incorporating self-care and peer support can empower clients and positively impact their relationships and quality of life [120, 156]. Most interventions in this review, in both transit and resettlement countries, were delivered in a group setting that offered peer support and general psychoeducation. A qualitative study involving 30 refugees from Burundi, Burma, Congo, Rwanda and Bhutan, resettled in the United States and receiving treatment for symptoms of depression, PTSD or anxiety highlighted the importance of culturally competent peer-centred programs. The authors recommended intervention programs providing group-based social support and addressing practical needs such as employment, language and literacy training and healthcare access [157].

### Religious/spiritual strategies

Religious and spiritual strategies included praying, fasting, reading religious texts, and attending religious gatherings. Whether self-generated or delivered through intervention, these practices instilled a sense of peace, relief, endurance and perseverance. These findings align with other research illustrating the benefits of culturally tailored psycho-spiritual interventions and religious practices for the mental health and wellbeing of refugees and migrants [158–163].

The Arab culture emphasises religious and spiritual traditions [130, 164]. Studies in the United States and elsewhere indicate that Christians who attend church services experience great support from fellow churchgoers and a sense of comfort from prayers [124, 165]. Similarly, within the Islamic faith, absolute submission to God’s will, regular prayer, observing Ramadan, Quran reading and expressing gratitude to Allah significantly increase happiness and inner peace [123, 125, 166, 167]. The daily prayers promote relaxation and alleviate symptoms of anxiety and stress [123, 168, 169].

A German study identified numerous challenges to practicing religious self-care [69]. Worshippers generally prefer attending mosques that cater to their specific Islamic sect [170, 171]. Muslim males are encouraged to perform the five obligatory prayers at the mosque, particularly, the Friday noon prayer [172]. Religious clerics have significant social standing within the Arab-Muslim community and may offer counselling to those dealing with mental disorders [152, 173]. However, clerics often lack formal training in counselling and may stigmatise mental disorders, viewing them as God’s punishment [173–175].

### Other strategies

Although psychological, social and religious/spiritual strategies were dominant in this review, a range of other self-care strategies were also identified. Participants practicing physical activity and exercise, expressive arts, and self-development activities reported increased confidence and motivation. Growing research demonstrates the physiological and psychological benefits of physical activity among refugees and migrants in diverse settings [176–178]. Arts-based interventions have effectively reduced symptoms of anxiety, stress and depression [179]. Alongside talk therapies, evidence-based psychological interventions incorporating arts-based cultural activities could be suitable for individuals seeking mental health support in the Arab culture, where stigma around mental illness is prevalent [141, 152]. Further research is needed on incorporating Islamic art and culture into mental health interventions [180].

Unstable economic circumstances increase the risk of PTSD and depression among refugees and migrants [23]. Interventions combining social and economic skill-building with mental health support are considered an emerging practice to enhance economic empowerment and mental wellbeing for refugee and migrant youth [117, 181]. Addressing structural barriers to social and economic participation as well as social determinants of health will benefit new arrivals of all age groups [182]. Besides culturally and linguistically appropriate mental health interventions, addressing uncertain visa status, racism and discrimination, financial difficulties and lack of resources is essential [183, 184]. Implementing visa reforms, enforcing fair migration and workplace laws, and adopting anti-discriminatory policies can enhance the overall wellbeing of refugees and migrants [17, 185].

### Implications for practice and service delivery

Specialist mental health services, which typically focus on treating individual patients, should consider group interventions incorporating self-care and peer support. For Arabic-speaking refugees and migrants, delivering these interventions in gender-specific groups that align with sociocultural norms can be beneficial for program engagement and outcomes. Supplementing standard psychological interventions with culturally relevant religious/spiritual strategies, social self-care and expressive arts-based strategies, as identified in this review, is also likely to be helpful.

Within the primary and community health context, collaborating with and training of religious leaders and community health workers on culturally and linguistically adapted mental health self-care strategies is beneficial for Arabic-speaking refugees and migrants. Investment in facilities to engage in religious practices, recreational activities, and female-friendly meeting spaces in refugee camps and resettlement countries is crucial. Integrating digital technology-mediated mental health self-care into primary healthcare services can potentially minimise psychiatric outpatient care, particularly in UMICs and LMICs [39, 149, 186]. Improving internet access, offering affordable mobile data packages, providing digital health literacy training, and incorporating clear instructions in local Arabic dialects for refugees and migrants with low education levels would enhance user experiences and the adoption of digital self-care strategies [149].

### Recommendations for research

Additional research is needed to explore mental health self-care strategies among Arabic-speaking migrants and refugees in Western resettlement countries, focusing on at-risk groups such as unaccompanied refugee minors, females, and older adults. Examining mental health self-care strategies among Arabic-speaking refugees and asylum seekers in low-income transit countries is also recommended. Studies in this review focused on Arabic-speaking migrants, refugees and asylum seekers aged 12 years and older. Given the significance of developmental factors in coping with stress [187], future reviews should consider adolescents and children separately from adults. Including a qualitative component in intervention studies would provide valuable insights into participant experiences and community perspectives. Promising areas for future research include the development of group interventions to facilitate social networks and the design, delivery, and further evaluation of guided psychological self-care apps. Further examination of the effectiveness, cultural appropriateness, and user experiences associated with physical activity, arts-based activities, and religious/spiritual practices as mental health self-care strategies is also warranted. Finally, further research is needed to better understand the relationship between various self-care strategies and measures of mental health and wellbeing, their effectiveness in alleviating symptoms of common mental disorders, and their impact on daily functioning within the target population in different contexts.

### Strengths and limitations

This review employed a rigorous systematic methodology and a comprehensive search strategy to examine the published and grey literature on mental health self-care strategies for Arabic-speaking refugees and migrants, including intervention and observational studies in English and Arabic. We adopted a broad definition of self-care, with implications for both public health and clinical practice [38]. The inclusion of observational studies provided a better understanding of self-generated strategies. All included intervention and observational studies were appraised as high or medium quality. Multiple reviewers independently screened and assessed the studies, including the five records authored by members of the research team, to remove selection bias. The inclusion of grey literature helped mitigate publication bias effects. Findings were reported according to the PRISMA guidelines [44, 56]. There are several limitations to note. Firstly, despite a comprehensive search strategy, it is possible that some potentially eligible studies may have been overlooked due to the vast scope of the concept definitions. Furthermore, papers published in languages other than English or Arabic may have yielded additional results. Moreover, in this review the target population was treated as a homogenous group, however cultural, religious and ethnic diversities within Arabic-speaking countries, along with differences in healthcare infrastructure across their countries of origin, may impact the generalisability of our findings to all Arabic-speaking individuals. The diversity of intervention studies, including variation in design, samples, intervention components, and outcome measures, made it challenging to determine the most effective and culturally appropriate self-care strategies. Despite the absence of diagnostic labels for participants, observational studies substantially add to the evidence of self-generated self-care practices. Given the known high prevalence of depression, anxiety and PTSD among Arabic-speaking refugees and migrants, these disorders are likely to be common in the study samples. Finally, most studies described multiple self-care strategies, making it difficult to reach definitive conclusions about the effectiveness of specific or groups of self-care practices.

## Conclusions

Refugees and migrants are more vulnerable to mental disorders than the general population yet are less likely to seek professional help or utilise mental health services. This review contributes to the expanding knowledge on mental health self-care for Arabic-speaking refugees and migrants. It describes how social, religious/spiritual, psychological and other self-care strategies provide meaningful and positive experiences for these populations and reinforces the effectiveness of culturally appropriate interventions in reducing symptoms. Insights from this review can be used to inform mental health interventions in diverse settings among this vulnerable population, adhering to the WHO service pyramid model, which has self-care as the basis for all other forms of care, including informal community care and formal services. Further research is needed in resettlement and transit countries to guide mental health service delivery and primary healthcare initiatives.

### Electronic supplementary material

Below is the link to the electronic supplementary material.

**Supplementary Material 1: Table 1-**Study characteristics.** Table 2-**Participant characteristics. **Table 3-**Intervention. **Table 4-**Effectiveness. **Table 5-**Cultural appropriateness. **Table 6-**Participant experience

**Supplementary Material 2:** PRISMA 2020 Checklist

## Data Availability

The authors confirm that the data supporting the findings of this study are available within the article [and its supplementary information files].

## List of abbreviations

CBPT: Cognitive Behavioural Play Therapy
CBT: Cognitive Behavioural Therapy
HIC: High-Income Country
JBI: Joanna Briggs Institute
LIC: Low-Income Country
PRISMA: Preferred Reporting Items for Systematic Reviews and Meta-Analyses
PTSD: Posttraumatic Stress Disorder
TRT: Teaching Recovery Techniques
UMIC: Upper Middle-Income Country
WHO: World Health Organization
